# Facile fabrication of linezolid/strontium coated hydroxyapatite/graphene oxide nanocomposite for osteoporotic bone defect

**DOI:** 10.1016/j.heliyon.2024.e31638

**Published:** 2024-05-22

**Authors:** Shuhui Wu, Yunxiao Lai, Xian Zheng, Yang Yang

**Affiliations:** a Department of Neurosurgery, Zhumadian Central Hospital, Zhumadian, 463003, China; b Medical College, Huanghuai University, Zhumadian, 463003, China; c Department of Obstetrics, Wenling First People's Hospital, Wenling, 317500, China

**Keywords:** Antibacterial, Graphene oxide, Hydroxyapatite, Linezolid, Osteoporotic bone defect, Strontium

## Abstract

Hydroxyapatite (HAp) coatings currently have limited therapeutic applications because they lack anti-infection, osteoinductivity, and poor mechanical characteristics. On the titanium substrate, electrochemical deposition (ECD) was used to construct the strontium (Sr)-featuring hydroxyapatite (HAp)/graphene oxides (GO)/linezolid (LZ) nanomaterial coated with antibacterial and drug delivery properties. The newly fabricated nanomaterials were confirmed by X-ray diffraction analysis (XRD), Fourier-transform infrared spectroscopy (FTIR), and X-ray photoelectron spectroscopy (XPS) analysis and morphological features were examined by scanning electron microscope (SEM) analysis. The results reveal multiple nucleation sites for SrHAp/GO/LZ composite coatings due to oxygen-comprising moieties on the 2D surface of GO. It was shown to be favorable for osteoblast proliferation and differentiation. The elastic modulus and hardness of LZ nanocomposite with SrHAp/GO/LZ coatings were increased by 67 % and 121 %, respectively. An initial 5 h burst of LZ release from the SrHAp/GO/LZ coating was followed by 14 h of gradual release, owing to LZ's physical and chemical adsorption. The SrHAp/GO/LZ coating effectively inhibited both *S. epidermidis* and *S. aureus*, and the inhibition lasted for three days, as demonstrated by the inhibition zone and colony count assays. When MG-63 cells are coated with SrHAp/GO/LZ composite coating, their adhesion, proliferation, and differentiation greatly improve when coated with pure titanium. A novel surface engineering nanomaterial for treating and preventing osteoporotic bone defects, SrHAp/GO/LZ, was shown to have high mechanical characteristics, superior antibacterial abilities, and osteoinductivity.

## 1 Introduction

Traumatic damage or illness-related sickness can seldom mend prominent segmental bone abnormalities independently [1]. The autologous transplant suffers a drawback because its status is the gold standard in treating major segmental bone lesions [[2], [3], [4]]. On the other hand, Allografts have limitations in source availability and the risk of immunogenic rejection, making them less suited for dealing with significant bone lesions [5]. As a result, to meet the high demand in clinics, a synthetic bone transplant alternative must be developed [6]. Bone graft substitutes that are ideal have biocompatibility, osteoconductivity, and mechanical qualities close to natural bone. Bioactive sites abound in natural biomaterials, which are also biocompatible in large quantities [7]. Because of this, they are frequently employed to simulate the extracellular matrix. One biomaterial with high biocompatibility is Chitosan when it comes to tissue engineering. To further boost osteogenic differentiation and cell adhesion, it has been shown to increase the activity of the ALP and stimulate cell proliferation [[8], [9], [10], [11], [12], [13]].

Osteoconductivity, osteoinduction, and delayed biodegradability in situ make it an excellent material for bone grafting [[14], [15], [16]]. Hydroxyapatite, replaced with an inorganic element (such as strontium, magnesium, and silicon), has a lower absorption rate than pure HA [17]. Hydroxyapatite (HA) is widely utilized as a ceramic biomaterial due to its ability to closely resemble the mineral composition of vertebrate bones [18]. Nevertheless, this biomimetic material exhibits inadequate mechanical characteristics, including diminished tensile and compressive strength, rendering it unsuitable for bone tissue engineering (BTE) [[19], [20], [21]]. Due to this rationale, hyaluronic acid (HA) is frequently employed in conjunction with other polymers and crosslinkers as composites to enhance the mechanical characteristics and overall efficacy of implanted biomaterials designed for orthopedic purposes [22]. Osteoblast activity and differentiation can be increased, whereas osteoclast formation and proliferation are inhibited. However, because of its weak mechanical properties and lack of durability, HAp cannot be used as a scaffolding material. Polymers, alumina, titanium alloys, and carbon nanostructures have been utilized to tackle this problem [[23], [24], [25], [26]].

Graphene oxide (GO), which possesses excellent biocompatibility and mechanical characteristics, is frequently employed in tissue engineering. Many oxygen-containing groups are found in this compound, including hydroxy, carboxyl, epoxy, and carbonyl groups [[27], [28], [29]]. These groups get GO hydrophilic and dispersible but act as nucleation sites for other nanomaterials. The surface structure of GO may be effectively coated with HAp nanomaterial and manage the accumulation of HAp on the surface to ensure that HAp grows evenly. As a result, combining GO and HAp can improve the mechanical strength and toughness of HAp, making it more suitable for bone replacement [30]. In addition, the sharp edges of GO nanosheets may directly disrupt the bacterial membranes, and the surface of GO can induce oxidative stress to kill bacteria, making them good antibacterial material. Given this description, GO may serve a new function in providing antibacterial capabilities to bone replacement scaffolds [31]. Additionally, GO has been shown to expedite the differentiation of hBMSCs into osteogenic cells and increase the vascularization of tissue-engineered bone [32]. Nie et al. observed that the integration of GO could increase the expression levels of vWF and ANG-1, which indicates the formation of vascular networks [33]. For tissue bone engineering, we looked at whether strontium-substituted hydroxyapatite (SrHAp) produced on an organic polymer nanosheet may better support bone regeneration [34].

In orthopedic and trauma surgery, infection of the bone and joint tissues is still one of the most severe concerns. *Staphylococcus aureus* (MRSA) and *Staphylococcus epidermidis* (MRSE) are two of the most common infections found in the human body [[35], [36], [37]]. Clinically, linezolid was effective for MRSA infections as usual vancomycin treatment [38]. Linezolid can be taken orally or intravenously(i.v.) and will have the same effect on the body. For antibiotics to be effective, they must be able to penetrate the bone and joint infection sites. During routine complete hip and knee replacements, the penetration of linezolid into noninfected bone, muscle, and hematoma fluid was recently assessed [39]. However, the penetration of linezolid into the infected osteoarticular tissues has not been examined. The bone and joint tissues of individuals with proven methicillin-resistant staphylococcal infections were thus tested for linezolid concentrations [40].

**Fig. 1:**
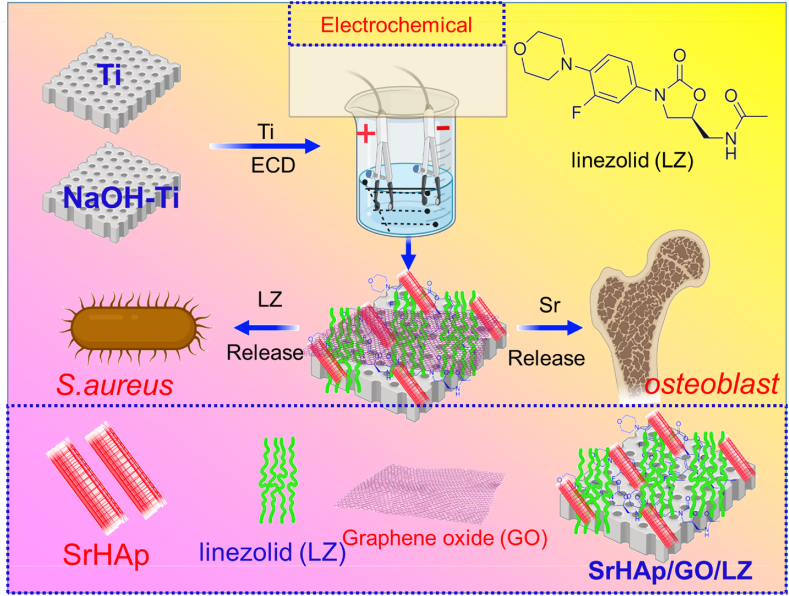
The construction method of SrHAp/GO/LZ multifunctional Ti surface coating improves the HAp coating's mechanical capabilities, antibacterial properties, and osteogenic activity in this study.

Osteoporosis is an extensive systemic disease considered by a reduction in bone imbalance and mass of the bone tissue. Clinical and experimental investigations keen to examining the pathogenetic mechanisms of osteoporosis exposed the vital role of epigenetic factors, cellular senescence, oxidative stress, inflammation, and estrogen deficiency in advance of bone resorption due to osteoclastogenesis, and bone formation and decreased mineralization of bone tissue to reduced function of osteoblasts triggered by age-depended differentiation and apoptosis of osteoblast ancestors into adipocytes. In this present investigation, electrodeposited (ECD) on the hot alkali-mediated titanium surfaces were utilized to deposit SrHAp/GO/LZ multifunctional coatings based on our past expertise with ECD hybrid HAp coatings. SrHAp/GO/LZ nanomaterial coating fabrication on Ti is demonstrated in Fig. 1. This examination successfully developed the microstructure surface, the effect of linezolid phase composition, in vitro cellular responses of composite coatings, the hydrophilicity of coated implants, and roughness on the antibacterial property of nanocomposite coatings.

## 2 Experimental section

### 2.1 Materials and fabrication method

Linezolid was obtained from Hisun Pharmaceutical (Zhejiang, China). Strontium, titanium, hydroxyapatite, and graphene oxide were obtained from Nanjing Emperor Nano Material Co. Ltd. (Nanjing, China). Osteoblasts MG-63 (School of Life Sciences, Southwest Jiaotong University) were grown in complete Minimum Essential Medium Alpha (MEM-, 10 % fetal bovine serum (FBS), 100 U/mL penicillin and 100 U/mL streptomycin (Thermo Fisher Scientific, Waltham, MA, USA). Osteoblast differentiation media was prepared by adding 10 mM sodium -glycerophosphate and 50 g/mL ascorbic acid to complete phenol red-free MEM-α. Then, 5 × 10^4^ cells/mL cells were seeded per substrate and placed in a 24-well plate.

Pure titanium (ASTM F67 Grade-2, 10 × 10 × 1 mm, 99.9 %) was utilized in the ECD method. Using suitable sandpaper, titanium was polished, soaked in 5 % hydrochloric acid for 1 min, then ultrasonic-assisted washed in methanol and aqueous medium for 5 min. A NaOH solution for 90 min at 80 °C produced a considerable quantity of TiOH on the surface of the Ti substrate, which was then dried in an oven at 65 °C for further processing.

The electrodeposition base liquid has the following formula (in mass fractions): The following substances were dissolved in 1000 mL of ultrapure water: 6.3 g of calcium nitrate, 0.90 g of M sulfate, and 2.88 g of NH_4_H_2_PO_4_. As shown in Fig. 1, an electrolyte with a 1 mg/mL concentration of linezolid was used to construct the SrHAp/LZ coating. As mentioned earlier, drops of the GO suspension were slowly added to the electrolysis to reach the desired 75 g/mL GO concentration for the SrHAp/GO/LZ coating. The temperature was lowered to 25 °C, and the electrolyte's pH was adjusted to 4.06. Three-electrode ECD devices were built using titanium sheets for cathodes, platinum sheets for anodes, and saturated calomel electrodes for reference. There was no change in the 2.5 cm distance between the cathode and anode electrodes. The electrolyte was agitated at 120 rpm during the ECD process to prevent Go's precipitation. After the experiment, samples were taken from the electrolyte and stored in a freezer.

### 2.2 Characterization method of SrHAp/GO/LZ coatings

The specimens were characterized by field emission scanning electron microscopy (FE-SEM, JSM7500F), energy dispersive spectrometer (EDS) and X-ray diffraction (XRD, X'Pert PRO, The Netherlands), Fourier transforms infrared spectroscopy (FTIR, Nicolet is5) in the range in between 500 and 4000 cm^−1^. The static water contact angle on the sample surfaces was determined by an angled instrument (DSA100, Krüss, Germany) according to the technique used in our previous research study. To assess the exact composition of the SrHAp/GO/LZ coating, thermogravimetric analysis (TGA) was used to characterize the composition of the SrHAp/GO/LZ coating quantitatively. TGA was carried out for each coating with a heating rate of 10 °C/min up to 700 °C TGA using a SETARAM Labys SDT Q600 Simultaneous Thermal Analyzer device under the nitrogen atmosphere.

### 2.3 Nanoindentation investigation

The Young's modulus and hardness of the SrHAp/GO/LZ were measured using a nanoindenter (TriboScope®, Hysitron Inc.). This method was utilized to conduct nanoindentation testing on the materials. To verify the assessment, a load-penetration depth curve was employed. The maximum load force was 6000 μN. The experiment was checked for correctness using 8–12 indentations for each sample [27].

### 2.4 Sr and linezolid release assessments

A 10 mL centrifuge tube combined the SrHAp, GO, and LZ mixtures. It was held at 37 °C by adding 1 mL phosphate-buffered saline. To maintain PBS fresh, it was collected at intervals of one day, three days, five days, eight days, eleven days, and fourteen days and kept at 4 °C. In this experiment, the Sr element concentration was detected using an inductively coupled plasma mass spectrometer (ICP-MS, Agilent 7700x, USA), and the release curve was displayed.

SrHAp/GO/LZ release kinetics rates, were measured according to previously described procedures. A 24-well plate with SrHAp/GO/LZ coatings was deposited in a shaker with 1 mL of PBS poured into each well and gently shaken (37 °C). Every 3, 5, 8, 11, and 14 h, fresh PBS was collected and kept at 20 °C. The LZ concentration was measured using a UV spectrophotometer. A standard calibration curve of LZ concentrations was employed to determine the concentration of LZ in PBS with time.

### 2.5 Antibacterial property

#### 2.5.1 Evaluation zone inhibition

Efforts to combat infection have employed *S. aureus* and *S. epidermidis* in the lab. It is typical for these microbes to be the causative agents of clinical bone and joint infections [41]. In pre-experiments, it was discovered that 81 % of the LZ in SrHAp/GO/LZ was released during the first 14 h, indicating an abrupt release. Bacterial infections develop early in surgery, according to clinical investigations. For this work, the antimicrobial time is set at 14 h. The bacterial suspension was adjusted to a concentration of 107 colony-forming units (CFU)/mL based on early studies. *S. aureus* and *S. epidermidis* was used to inoculate each plate with bacteria, and the antibacterial activity was measured by measuring the area of the zone of inhibition (ZOI). During the investigation, Ti samples coated with SrHAp/LZ and SrHAp/GO/LZ were designated as experimental and control groups. The antibacterial studies disinfected all glassware and associated tools on SrHAp/GO/LZ. Antibacterial tests were conducted on a random sample pool of three individuals.

#### 2.5.2 Spread plate test

In colony counting tests, bacteria with a 10^7^ CFU/mL concentration were utilized as a standard. A glass tube containing 800 μL of Luria-Bertani (LB) was filled with 100 μL of the bacteria solution. The samples were placed in a centrifuge tube containing microbial solutions and incubated in a bacterial incubator at 14 h. It was then scraped out of the well plate, equally spread over the Agar plate, and incubated for 24 h at 37 °C in an incubator at an appropriate temperature. Finally, each plate had its colonies imaged and numbered [[42], [43], [44]].

### 2.6 Osteoblast culture and evaluation

#### 2.6.1 MG-63 proliferation

Cell proliferation of MG-63 on all the substrates was assessed on day 1, day 3, and day 5. Cells were incubated in Cell Counting Kit-8 working reagent, prepared by mixing CCK-8 in complete MEM-(1:10 v/v). After 4 h, the absorbance was measured at 450 nm by a microplate reader (BioTek Instruments, Inc. VT, USA).

#### 2.6.2 ALP activity, Collagen-1 and osteocalcin production of MG-63 cells

The Alkaline Phosphatase Assay Kit measured ALP activity in the cell culture supernatant of osteoblasts at different periods (day 3, day 5, and day 14). (BioAssay Systems, Hayward, CA, USA). Briefly, 50 μL of the sample was incubated for 4 min with 150 μL of ALP working reagent. A microplate reader measured 405 nm absorbance at 0 and 4 min (Synergy H1). The total protein was calculated using the kit's method to normalize the ALP activity in terms of pNP generated. On day 14, we assessed collagen 1 and osteocalcin in the cell culture supernatant using the Pro-Collagen I alpha 1 Simple Step ELISA kit (Abcam, Cambridge, UK) and the Osteocalcin ELISA kit (Cloud-Clone Corp., Katy, TX, USA), respectively, and the data were adjusted to total protein [29,33,45].

## 3 Results and discussion

### 3.1 Characterization of SrHAp/GO/LZ

**Fig. 2:**
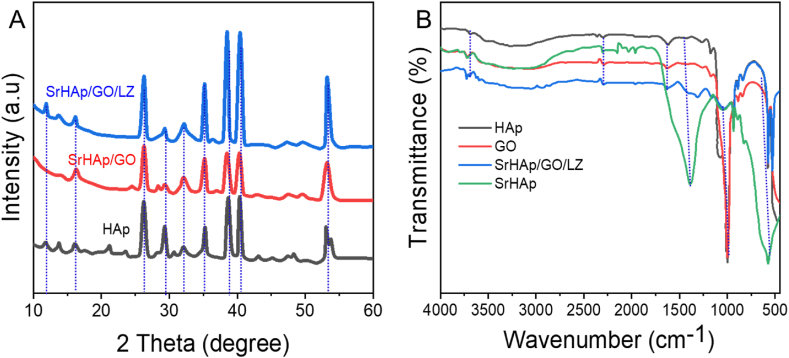
A) X-Ray diffraction analysis (XRD) spectra of HAp, SrHAp/GO, and SrHAp/GO/LZ. B) Fourier-transform infrared spectroscopy (FTIR) spectral analysis of Hap, GO, SrHAp, and SrHAp/GO/LZ.

XRD measurements revealed the HAp, SrHAp/LZ crystal structures, and SrHAp/GO/LZ (Fig. 2A). The Ti substrate coat was discernible because of the coating's nondense structures. All peak was consistent with the HAp crystal diffraction peak, excluding the titanium peak. It was determined that 2ɵ values between 26.1° and 31.9° corresponded to planes (002), (210), (211), (310), (222), (213), and (004) on the XRD PDF card of pure HAp (PDF No-09-0432), while the diffraction peaks on the three curves agreed with those on the card at 2ɵ values between 31.9° and 53.4°. The 11.6° diffraction peak was also matched with CaHPO_4_.2H_2_O (HAp, PDF # 03–0836). However, no crystal GO diffraction peaks have been found, which might result from a disorderly three-dimensional arrangement of GO atoms or the destruction of GO's crystal structure [46]. Because of the width of the HAp coating (14 μm) and the thinness of the sodium titanate nanocoating on the surface of Ti, the diffraction peak of sodium titanate was not identified.

Fig. 2B depicts the FT-IR spectral analysis of pure HAp, GO, SrHAp/LZ, and SrHAp/GO/LZ nanocomposite coatings. GO's FTIR results showed a prominent peak among 2801 cm^−1^ and 3512 cm^−1^ that belonged to OH^−^ groups. It's easy to identify epoxy by its distinctive adsorption peaks at 841 cm^−1^. For the first time, oxide functional groups in GO were established by two distinct C–O peaks centered at 1399 cm^−1^ and 1059 cm^−1^. The occurrence of oxygen-featuring compounds in the GO-founded FT-IR spectra verified the complete oxidation of graphite. As shown in Fig. 2B, the infrared peak locations for each of the three coatings were 561, 601, 974, 1029, and 1099 cm^−1^. The PO_4_^3−^ peak in the HAp structure was assumed to be the source of these distinctive peaks. HPO_4_^2−^ in the calcium dihydrogen phosphate phase is the primary cause of the distinctive peaks with 527 cm^−1^, 923 cm^−1^, and 1108 cm^−1^. The –OH peak from HAp occurred at 636 cm^−1^. Three coatings produced in this study were found to have similar overall profiles and molecular vibration properties when analyzing their FTIR spectra. CO_3_^2−^ peaks (879 cm^−1^, 1411 cm^−1^, and 1490 cm^−1^) from SrHAp/GO/LZ coatings were visible, unlike the HAp and SrHAp coatings. Water absorbs carbon dioxide from the atmosphere during the sample preparation procedure, accounting for most of the CO_3_^2−^ ions. When CO_3_^2−^ replaces the PO_4_^3−^ in HAp [Ca_10_(PO_4_)_6_(OH)_2_]. If carbonated HA (CHA) is found in human bone, we have a strong case for using it as a biomimetic implant covering. SrHAp/GO/LZ composite coating FTIR showed two soft peaks at the wavenumber of 2855 and 2925 cm^−1,^ which were the CH_2_ peaks, respectively.

**Fig. 3:**
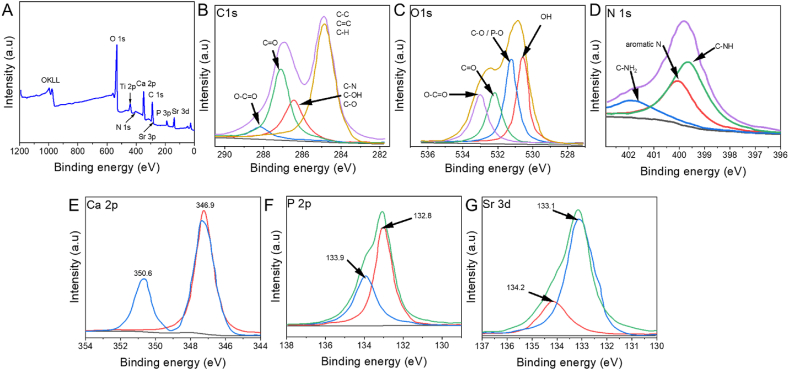
A) X-ray photoelectron spectroscopy (XPS) collected for SrHAp/GO/LZ. High-resolution spectra of B) C 1s. C) O 1s. D) N 1s. E) Ca 2p. F) P 2p. G) Sr 3d.

The chemical valence of the SrHAp/GO/LZ composite coating was discovered via XPS spectrum analysis. On the SrHAp/GO/LZ composite, the Sr3d, Sr3p, N1s, C1s, O1s, Ca2s, and P2s peaks were observed. Fig. 3A–G shows the HR spectral of each element to understand its valence state. Deconvoluting the high-resolution Ca2p spectra (Fig. 3E) revealed two distinct peaks at 347.9 eV and 352.4 eV, determined to be Ca2p3/2 and Ca2p1/2, respectively. Two firm peaks occurred at 133.7 eV and 134.1 eV, which matched P 2p3/2 and P 2p1/2 when the high-resolution P2p spectra (Fig. 3F) were deconvoluted. Fig. 3G shows the deconvoluted Sr 3d high-resolution spectra, revealing two notable peaks at 133.9 eV and 132.9 eV, respectively, resulting from Sr3d3/2 and Sr3d5/2. Simultaneously, the N1s peaks were tailored, agreeing to N–H and N–C at 398.7 eV and 401.2 eV, respectively (Fig. 3). Interestingly, a faint signal at 402.1 eV was fitted, which shows that N1s, suggesting that LZ has coated onto the GO surface, complete the CN^3+^ group. Identifying HAp and Linezolid using Ca2p and N1s as important components is possible. When the N1s peak in the XPS is combined with the Sr3d and S33p signals, the Sr^2+^ and LZ have effectively incorporated into the HAp structure. Four satellite peaks may be deconvolved for high-resolution spectra of O1s (Fig. 3C), namely OH at 531.1 eV, C–O/P–O at 533.4 eV, C=O at 533.2 eV, and O–C=O at 534.0 eV. The high-resolution spectra of C 1s (Fig. 3B) may also be separated into four satellite peaks: O– C–C/C–H/C=O at 285.7 eV, and C–O/C–N/C–OH at 287.3 eV, C=O at 288.2, and C=O at 289.0 eV. They also agreed with the crystalline structure of linezolid and graphene oxide. The C=O and C–O groups can electrostatically adsorb Ca^2+^ to the HAp in the ECD approach. This occurs during the HAp synthesis. Adsorption of Ca^2+^ in HAp is possible via strong chelation of the –C=O group. Overall, XPS data may be an excellent foundation to include SrHAp/GO/LZ composite coatings with positive outcomes.

**Fig. 4:**
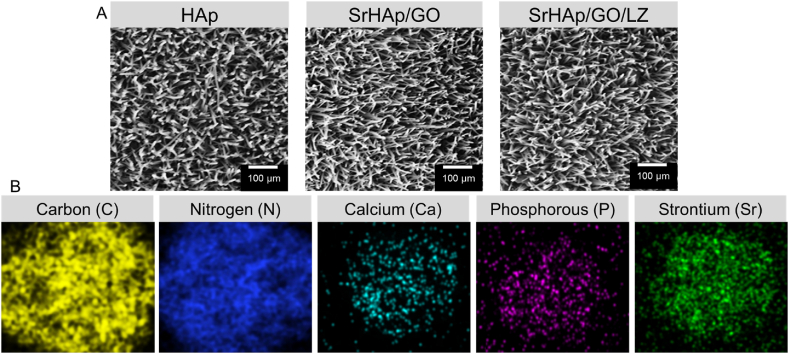
A) scanning electron microscope (SEM) images of the HAp surface, SrHAp/GO surface, and SrHAp/GO/LZ surface. B) EDS spectra of HAp, SrHAp/GO, and SrHAp/GO/LZ. EDS mapping spectrum of SrHAp/GO/LZ.

SEM images of HAp, SrHAp/LZ, and SrHAp/GO/LZ coatings are shown in Fig. 4A. Nanowhisker pins or flakes were visible on the surface of the pure HAp coating (Fig. 4A). Fig. 4A shows that SrHAp and SrHAp/GO/LZ coatings have smoother surfaces. The porosity of the coating reduced when Sr and LZ were added, resulting in a coating with a layered crystal morphology. Doping GO with SrHAp/GO/LZ decreased the crystallite size of the coating, agglomerated and firmly linked certain crystals, and eliminated most of the nanowhisker structures. According to past studies [47], the surface of biomaterials with nanostructured modifications can better regulate osteoblast function.

SrHAp/GO/LZ coating's elemental composition can only be determined using EDS to identify valence states within 10 nm of a layer's thickness (Fig. 4B). Only C, O, P, and Ca components were found on HA-coated surfaces (Fig. 4B). Two additional Cl and N peaks in the SrHAp/LZ coating were compared to the pure HAp control group, indicating that LZ was effectively merged into the nanocomposite coatings. Sr^2+^ has been doped into SrHAp/LZ, resulting in the Sr peak, and the SrHAp/GO/LZ and SrHAp/LZ are nearly identical (Fig. 4B). This may indicate that the GO was successfully absorbed into the composite coating, given the concentration of C in SrHAp/GO/LZ is higher than in SrHAp/LZ. This composite coating's EDS mapping indicated a uniform distribution of the components it contained. N, C, O, P, and Ca were uniformly dispersed on the nanocomposite surface (Fig. 4B).

Titanium surfaces that have been treated with hot alkali are susceptible to forming TiOH groups; Ca^2+^ may bind to the titanium surface's hydroxyl groups and TiOH groups and construct HAp crystals in a GO-free electrolyte. Increasing titanium's surface pH will prime the COOH deprotonation, resulting in GO surface negative charges during the electrolytic deposition. HAp crystal nuclei are formed via electrovalence bonding between Ca^2+^ ions and the PO_4_^3−^ ions around them. More potential nucleation of HAp crystals is available because of the GO's 2D structure. SrHAp/GO/LZ naturally generates smaller nanostructures than HA. The 2D structure of GO may have been covered with HAp crystals on its surface, making it impossible to identify using SEM images (Fig. 4A). On the other hand, linezolid has an alkaline molecule and, when dissolved in an electrolyte, will have a positive charge (isoelectric point = 8.3). Positive charge antibiotics will agglomerate on the Ti surface under the influence of the electric field force, which will speed up the rise in pH at the cathode and aid in the deposition of HA. SrHAp/LZ coatings, on the other hand, are denser because of the help of linezolid. It is also possible to use the –NH_3_ of LZ to remove the PO_4_^3−^ ions from GO and transport them to the surface so HAp crystals can grow. A denser whisker-integrated nanostructure is formed on the titanium's surface when LZ and GO are introduced to the electrolyte. This increases mechanical strength and adhesion, essential properties for a SrHAp/GO/LZ coating.

**Fig. 5:**
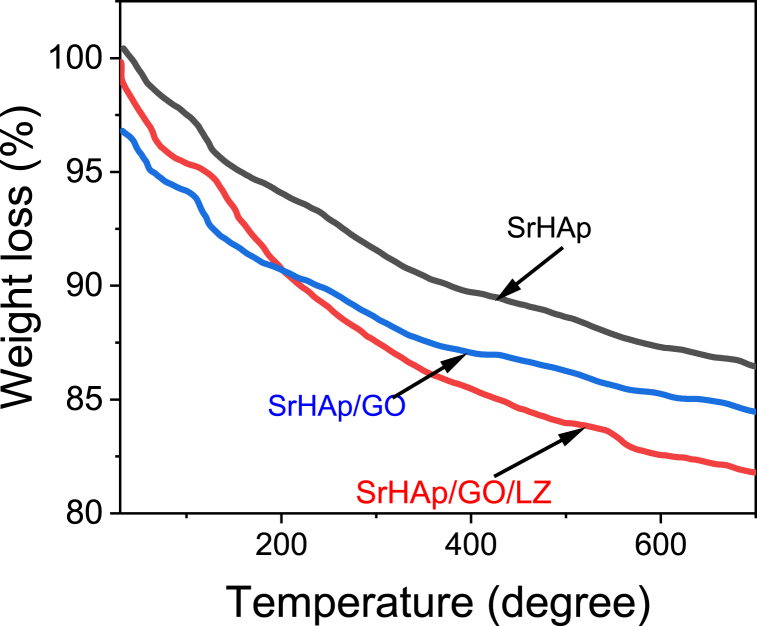
Thermal gravimetric analysis (TGA) analysis of SrHAp, SrHAp/GO, and SrHAp/GO/LZ coatings.

The SrHAp/GO/LZ coating cross-sectional image is depicted in Fig. 4A. The SrHAp/GO/LZ coating's cross-sectional thickness was around 15 mm thick. The coating's whiskers were all joined to form a single mass that was thick and homogeneous in appearance. It's not much of a difference between the thickness of HAp and SrHAp/LZ and SrHAp/GO/LZ. As observed in the section, the SrHAp/GO/LZ coating has a higher density than either HAp or SrHAp/LZ. In prior studies, electrodeposited HAp coatings have been reported to have a thickness between 10 and 70 μm; this value matches the apatite thickness in this study. The SrHAp, SrHAp/GO, and SrHAp-GO/LZ coatings were heated to 700 °C at 10 °C per min under a nitrogen environment (Fig. 5). All samples had a mass loss of 4 % at 100 °C due to the elimination of adsorbed water. A moderate and minimal weight loss during the heat treatment from room temperature to 700 °C, attributed to SrHAp's thermal solid stability.

### 3.2 Mechanical behavior of the nanocomposites

**Fig. 6:**
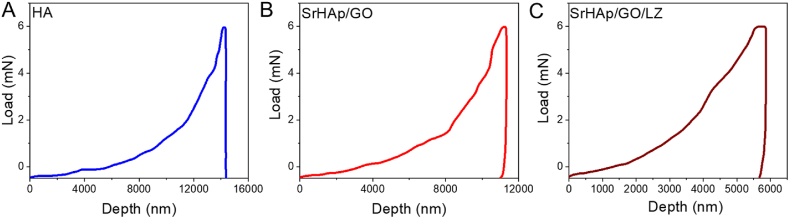
The nanoindentation examination achieved load-penetration depth curves for the HAp, SrHAp/GO, and SrHA/GO/LZ coatings.

Mechanical stability is critical to the coating's performance and longevity. In recent years, people have also considered increasing the mechanical characteristics of materials while inventing new biocompatible coatings. Nanoindentation analysis obtained the load-penetration depth curves of HAp, SrHAp/LZ, and SrHAp/GO/LZ by nanoindentation analysis, as shown in Fig. 6A–C. HAp, SrHAp/LZ, and SrHAp/GO/LZ had an elastic modulus and hardness of 1.4, 1, and 3, respectively. With the 6 mN load, the penetration depths of the films are around 14405 nm, 11,332 nm, and 5866 nm for the HAp, SrHAp/LZ, and SrHAp/GO/LZ films correspondingly. The lower the penetration depth, the better the coating's mechanical qualities. Coatings made using SrHAp/GO/LZ are the most pressure-resistant. Doping Ca–P with GO has been shown to improve the hardness and elastic modulus of the nanomaterial. Hardness and elastic modulus were raised by about 68.1 % and 122 %, respectively, when GO was included in this study's SrHAp/GO/LZ coating. The superior GO surface area 2D structure leads to a more significant SrHAp/GO interface volume ratio in the SrHAp/GO/LZ. GO's enhanced mechanical characteristics may be efficiently transmitted to HAp in this case, improving the mechanical properties of SrHAp/GO/LZ even more. The SrHAp/GO/LZ coating also decreases stress shielding.

### 3.3 Concentration Sr^2+^ and LZ release

**Fig. 7:**
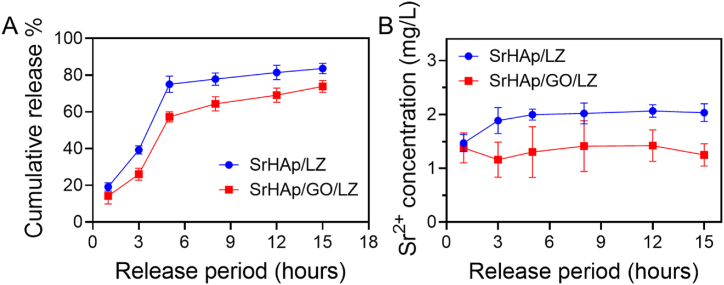
A) Linezolid release from the SrHAp/GO and SrHAp/GO/LZ coating. B) In vitro release profiles of Strontium from SrHAp/GO and SrHAp/GO/LZ coating.

The material's typical LZ release profiles exclusively utilize covalent bonds or van der Waals forces to link the medication to the substance. Toxicities in non-cancerous cells and tissues near the implants can occur because of the quick drug release rate and huge doses of medication released into the body. Coatings of SrHAp/LZ and SrHAp/GO/LZ were analyzed for LZ release characteristics. Fig. 7A showed that the SrHAp/LZ and SrHAp/GO/LZ coatings had similar kinetic curves of controlled release. The first quick release and the latter gradual release mode are commonly thought to represent the two phases of a drug-loaded material's release profile. Linezolid's release curve was found to follow the rule stated above. Fig. 7B shows that 71 % of LZ was released from the SrHAp/LZ coating during the first 5 h. The percentage of LZ released from SrHAp/GO/LZ was 55 %. This material releases many antibiotics into the body in just 5 h after implantation, giving it ample time to remove any biofilm that may form on the implant surface. After 14 h, the SrHAp/LZ and SrHAp/GO/LZ coatings released 81 % and 70 % of the LZ they contained. The SrHAp GO/LZ coating has an advantage over SrHAp/LZ when controlling LZ release. The chemisorption of the GO surface may explain why LZ is released from the SrHAp/GO/LZ coating at a sluggish rate. LZ may be strongly adsorbed by the GO surface, which comprises several oxygen-containing groups. These oxygen-containing groups can form covalent connections with LZ (Fig. 7B). However, it was found that the Sr^2+^ release rate from the SrHAp/LZ coating was greater than that of the SrHAp/GO/LZ coating. The significant GO adsorption may have something to do with this. Sr^2+^ ions were gradually and constantly released from SrHAp/GO/LZ in this investigation. SrHAp/GO/osteogenic LZ's impact can be considerably enhanced by adding this bone-promoting ingredient to the mix.

### 3.4 In vitro antibacterial functions

**Fig. 8:**
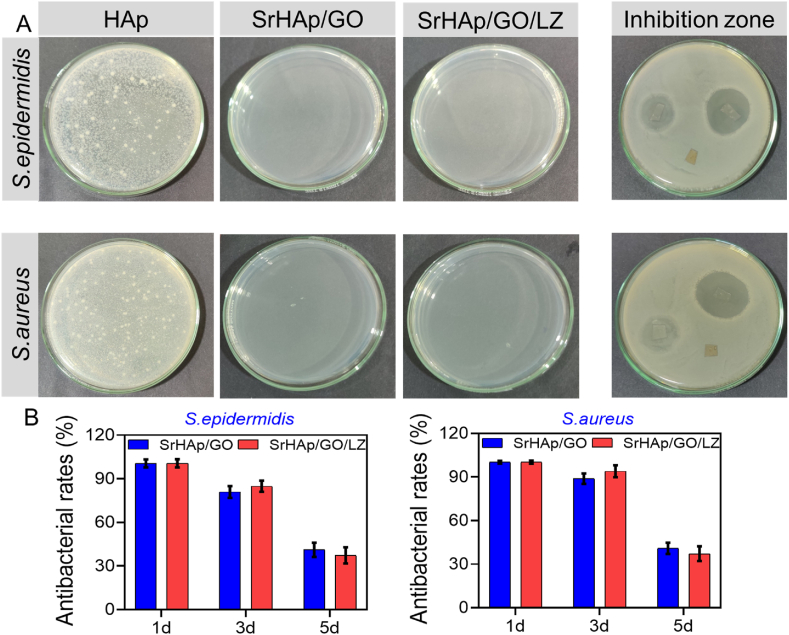
A) Inhibition zone image of *S. epidermidis* and *S. aureus* after 14 h of incubation; Typical images of cultivated *S. epidermidis* and *S. aureus* colonies from the specimens after 14 h of incubation. B) Long-term antibacterial rates of SrHAp/GO and SrHAp/GO/LZ against *S. epidermidis* and *S. aureus* after immersion for 1, 3, and 5 days.

Studies show that infections caused by Staphylococcus germs (most often Staphylococcal epidermidis) cause a growing number of failed implantations yearly, resulting in physical discomfort for the patients and financial hardship for their families. A substantial dose of antibiotics given early in colonization is the most effective strategy to keep biofilms from forming. Fig. 8A depicts the nucleic acid findings of the colony formation unit and zone of inhibition during the first 14 h of bacterial growth (Fig. 8B). SrHAp/LZ and SrHAp/GO/LZ loaded with linezolid prevented bacteria growth, and no colony was shown. Both can achieve a bacteriostatic rate of 100 % (Fig. 8B). In fact, despite the lack of linezolid, several colonies continued to flourish in HA. The inhibition zone experiment's findings revealed no zone of inhibition around the control group (HAp). SrHAp/GO/LZ and SrHAp/GO/LZ were found to have large bacteriostatic rings around them. Fig. 8A shows that the antibacterial properties of LZ-containing apatite coatings (SrHAp/LZ and SrHAp/GO/LZ) may endure for at least three days, as demonstrated by these results (Fig. 8B). However, after five days, the LZ-containing composite coating's antibacterial rate was drastically reduced to around 40 %. The antibacterial rate has gradually decreased because the LZ included in the coatings has been released after three days. Linezolid, the antibiotic we employed in this work, can efficiently prevent the formation of bacterial walls and kill many bacteria resistant. It is also routinely used in infected bone defects. If bacteria cannot acquire resistance to LZ, it is unlikely that they will cross-resistance. SrHAp/GO/LZ coatings demonstrate similar antibacterial properties to linezolid-containing HAp coatings.

**Fig. 9:**
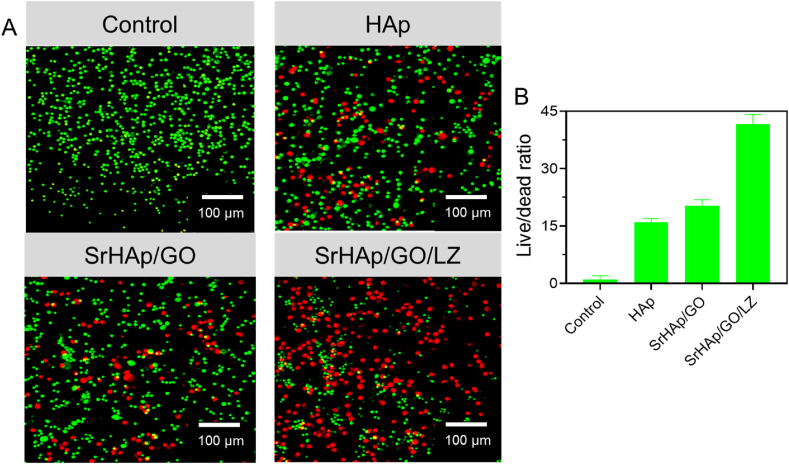
A) Live/dead image of control (untreated), HAp, SrHAp/GO, and SrHAp/GO/LZ against *S. aureus*. B) Live/dead percentage of control (untreated), HAp, SrHAp/GO, and SrHAp/GO/LZ.

Live/dead fluorescence staining was used to determine the coating's effect on bacterial adherence, and the results are presented in Fig. 9A. Both living and dead bacteria had damaged cell membranes colored red and green, respectively. Antibacterial and killing abilities were precise on the surfaces of SrHAp since there were virtually no living bacteria. HAp and SrHAp/GO/LZ coatings had a lot of green patches on their surfaces, indicating that their capacity to remove *E. coli* was insufficient. SrHAp and SrHAp/GO/LZ coatings for *S. aureus* had nearly no green patches on the surface, suggesting that most bacteria had perished. It was found that the SrHAp GO/LZ coating was more vulnerable to *S. aureus* than the prior results of antibacterial rate (Fig. 9B).

### 3.5 Biocompatibility and differentiation

**Fig. 10:**
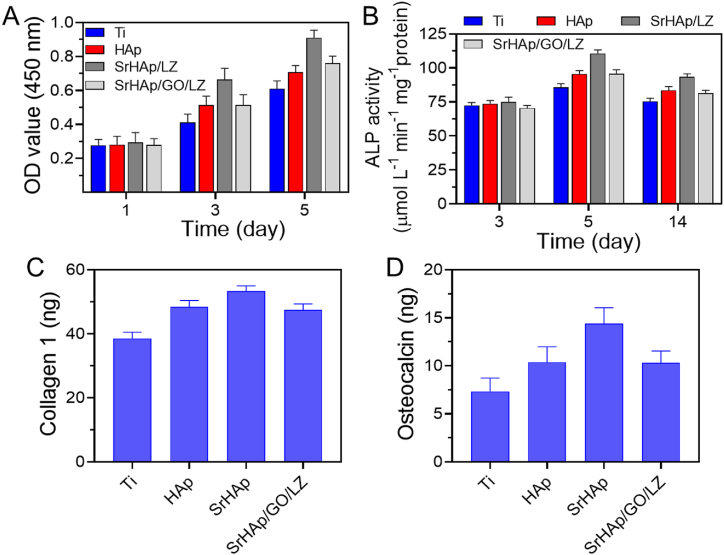
A) Cell proliferation and differentiation assessment of MG-63 cells cultured on the Ti, HAp, SrHAp, and SrHAp/GO/LZ samples by CCK-8 assay. B) ALP Activity. C) Collagen 1. D) Osteocalcin production of MG-63 cells.

Implant materials coating must be cytocompliant, which can stimulate bone growth without triggering the body's immune system. Cellular Proliferation on the Ti, HAp, SrHAp, and SrHAp/GO/LZ coatings was measured using the CCK-8 osteoblast experiment. Cell growth was not significantly different between the three coatings and Ti after one day of the culture of cell culture. After 3 and 5 days of culture on HAp, SrHAp, and SrHAp/GO/LZ, MG-63 cells proliferated substantially more than Ti (Fig. 10A). Cell growth investigations with other types of HAp coatings yielded comparable findings to these. SrHAp coatings were much more effective in stimulating osteoblast proliferation when cultivated for 3 and 5 days, demonstrating that Sr has a vital function. The SrHAp/GO/LZ coating and the HAp coating did not differ significantly. LZ and GO had no detrimental influence on the cell proliferation property on the apatite surface. The linezolid dosage utilized in this investigation didn't cause cytotoxicity in MG-63 cells.

Osteocalcin is a protein marker for later differentiation in MG-63 cells, while COLL 1 and ALP are indicators for early differentiation. ALP activity in MG-63 cells is shown in Fig. 10B. ALP activity in osteoblasts was not significantly different in the four samples after three days of culture. On day 5, ALP activity was at its maximum point. There was a considerable increase in ALP activity on HAp, SrHAp, and SrHAp/GO/LZ compared to Ti. SrHAp substantially increased ALP activity at 5 days compared to HAp and SrHAp/GO/LZ, showing that SrHAp was the most dominant activator of early osteogenic differentiation. The ALP activity of all four samples decreased after 14 days. SrHAp/LZ/GO had considerably greater ALP activity than HAp, SrHAp, and Ti, although this difference was not statistically significant. SrHAp's activity was greater than HAp and SrHAp/GO/LZ. Osteoblast differentiation is aided by ALP, which is most active in the early phases. Before the mineralization of the cellular matrix, ALP activity reduces dramatically.

A further 14 days of culture with SrHAp/GO/LZ exposed to osteoblasts allowed for analysis of alterations in osteocalcin and COLL 1 (a late differentiation marker). A statistically significant difference was found between MG-63 cells grown on SrHAp, SrHAp/GO/LZ, and Ti for the formation of osteocalcin (Fig. 10C). Like ALP findings, SrHAp-grown MG-63 cells secreted greater COLL 1 and osteocalcin levels than those grown on HA. SrHAp, on the other hand, offers the most evident benefit in inducing the differentiation of MG-63 cells. COLL 1 and osteocalcin content in HAp and SrHAp/GO/LZ were not significantly different. Accordingly, the results show that LZ/GO has no harmful impact on the differentiation of MG-63.

Cytocompatibility can be impacted by various variables, although SrHAp/GO/LZ exhibits high cytocompatibility. Sr, an essential trace element in human bones, has significantly impacted differentiation and cell proliferation (Fig. 10D). Additionally, investigations have demonstrated that micro/nanostructures on the surface of biomaterials may control the osteogenic effects of fibroblasts and osteoblasts. A clear advantage in osteoblast development may be shown once osteoblasts with nanowhisker are placed on the surface. When comparing SrHAp/GO/LZ and SrHAp/LZ, it is possible to conclude that the variation in surface topography has less influence on osteoblasts than previously thought. There is little doubt that strontium has a higher impact on cell proliferation and differentiation than any other element.

GO's hydrophilic functional groups, such as hydroxyl, epoxy, carboxyl, and carbonyl groups, are found on its surface, which means that SrHAp/GO/LZ containing GO may be more compatible with cells. Previous studies have used various cells to examine the adhesion and proliferation properties of graphene-enhanced HAp composites. MG-63 was shown to have high cell compatibility, consistent with our findings. Although GO's role in this process is well established, cell differentiation and signal transduction pathways are still unclear.

Finally, linezolid was chosen as the model medication because of its reduced cytotoxicity and higher cell compatibility with osteoblasts compared to other antibiotics. Linezolid-containing biomaterials and cytocompatibility may kill S.aureus with MG-63 cells (human osteoblasts). GO and linezolid was included in the coating simultaneously in this investigation. Using a ternary system design, it is possible to achieve high mechanical qualities and antibacterial and osteogenic activities in a coating. These three benefits are unrelated and don't affect one another. Animals will be implanted with the composite covering in later experiments to examine the nanomaterial's ability to mend infected bone deformities.

## 4 Conclusions

In this study, we effectively constructed a SrHAp/GO/LZ multifunctional Ti surface coating to improve the HAp coating's mechanical capabilities, antibacterial properties, and osteogenic activity. We used the ECD approach to include Sr, GO, and LZ into our standard HAp coating. Mechanical studies show that when GO was introduced into the coating, the SrHAp/GO/LZ coating hardness and elastic modulus rose from 27.3 to 45.7 MPa and 1.5–3.2 GPa, respectively. *S. aureus* and *S. epidermidis* demonstrated substantial antibacterial benefits within three days of exposure to the SrHAp/GO/LZ coating. This might help prevent biofilms from forming on the surface. According to cytology studies, cell adhesion, proliferation, and differentiation are better supported by SrHAp/GO/LZ than pure Ti. In addition to its hydrophilicity and roughness, the SrHAp/GO/LZ coating may also release Sr^2+^ ions. Excellent cell compatibility can be achieved through the combined effects of these elements. Due to its coordinated mechanical, antibacterial, and osteogenic properties, SrHAp/GO/LZ modified Ti may one day be employed to treat osteoporotic bone defect.

## Data availability statement

Not applicable.

## Financial & competing interests disclosure

This research received no specific grant from funding agencies in the public, commercial, or not-for-profit sectors.

## CRediT authorship contribution statement

**Shuhui Wu:** Writing – original draft, Formal analysis, Data curation, Conceptualization. **Yunxiao Lai:** Validation, Software, Methodology, Investigation, Formal analysis, Data curation. **Xian Zheng:** Writing – review & editing, Writing – original draft, Supervision, Formal analysis, Conceptualization. **Yang Yang:** Writing – review & editing, Writing – original draft, Visualization, Validation, Conceptualization.

## Declaration of competing interest

The authors declare that they have no known competing financial interests or personal relationships that could have appeared to influence the work reported in this paper.
